# Chemiluminescence detection of reactive oxygen species in isolated Kupffer cells during phagocytosis of *Treponema pallidum*

**DOI:** 10.1186/1476-5926-2-S1-S41

**Published:** 2004-01-14

**Authors:** Rita Aldini, Antonella Marangoni, Massimo Guardigli, Vittorio Sambri, Lorenzo Giacani, Marco Montagnani, Aldo Roda, Roberto Cevenini

**Affiliations:** 1 Istituto di Scienze Chimiche, University of Bologna, via S Donato 15, 40126, Bologna, Italy; 2 Sezione di Microbiologia, DMCSS, University of Bologna, Policlinico S. Orsola, via Massarenti, 40138 Bologna, Italy; 3 Dipartimento di Scienze Farmaceutiche, Univversità of Bologna, via Belmeloro 6, 40126, Bologna, Italy; 4 Dipartimento di Medicina Interna e Gastroenterologia, Policlinico S Orsola, via Massarenti 9, 40138, Bologna, Italy

## Introduction

Luminol amplified chemiluminescence is widely used for the sensitive detection of reactive oxygen species (ROS) production in cells such as polymorphonuclear leucocytes and macrophages [1]. However, little work in this field has been done in Kupffer cells (KC). Cellular immunity is primarily responsible for *Treponema Pallidum *clearance and resolution of the early lesions [2]. However, phagocytosis proceeds much slower than with other bacteria [3]. Activated Kupffer cells trigger a process called the respiratory burst, involving an increase in cellular oxygen consumption and the production of ROS, which are cytotoxic for a variety of microorganisms and contribute to deterioration of hepatic circulation. At present, there are not studies in the literature about the phagocytosis of *T. pallidum *by Kupffer cells and about the ability of the spirochete to induce the production of ROS by KC. In this study, we analyzed the interaction of *T. pallidum *with isolated rat Kupffer cells *in vitro*.

## Methods

### Bacterial strains and culture conditions

*T. pallidum*, Nichols strain, (purchased from Statens Serum Institute, Copenhagen, Denmark) was maintained by testicular passage in adults male New Zealand white rabbits.

### Separation of Kupffer cells (KC)

Kupffer cells were harvested following Smedsràd and Pertoft [4] with some modifications. The rat livers were perfused with collagenase, 0.05% in Hanks balanced solution. The KC were harvested following Percoll gradient separation and serial centrifugations. A 0.5-ml portion of cell suspension was added to 8-wells culture plate (LAB-TEK, Nalge-Nunc International, Naperville, Ill.). Macrophages were selected by allowing them to adhere for 2 hours at 37 degrees C, in an atmosphere with 5% CO_2_. Adherent cells were incubated with RPMI-1640 for 24 hours before performing the assays. More than 95% of adherent cells were esterase positive.

### Chemiluminescence detection of ROS production

Kupffer cells, isolated as above described, were suspended in culture medium and seeded (7 à l0^5 ^cells/ml) in plates for cell culture. After 24 hours, the culture medium was replaced with 200 microliters of fresh one, and 50 microliters of a 3 mM luminol solution in TRIS buffer (0.1 M, pH 7.4) were added to each well followed by 50 microliters of a spirochete suspension in culture medium. The final luminol concentration was 0.5 mM. A fixed concentration (0.8 micromolar) of phorbol myristate acetate (Sigma) was used as control in each run. The microtiter plate was placed in a luminometer at 37 degrees C, and the CL emissions, expressed as relative light units, were acquired at 2-minutes intervals for three hours, using an integration time of 10 s.

Quantitative evaluation of the signals was performed by integrating the CL signal over the whole measurement period; the CL response of the system was then expressed as the ratio between the integrated CL signal of the sample and that of the control. Additional experiments, in which no luminol was added, were performed as controls (these experiments didn't show CL signal either in the presence or in the absence of stimulating agents).

## Results

### Reactive oxygen species production by Kupffer cells following incubation with spirochetes

**Figure 1:**
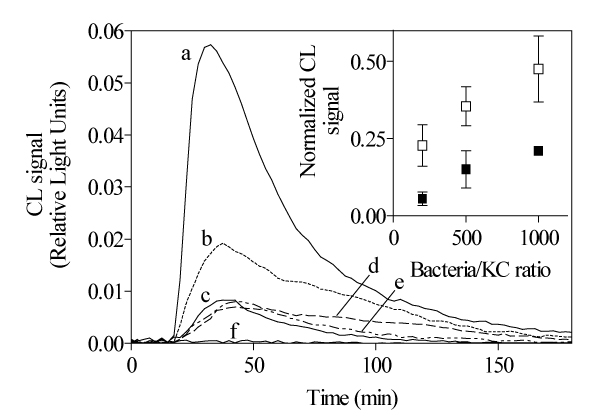
Generation of ROS by Kupffer cells incubated with various preparations of *T. pallidum*. Cells were incubated with live (e), heat-inactivated opsonized (b), heat-inactivated (d), or live opsonized (c) spirochetes, as well as with phorbol myristate (a). In the inset, dose-response curves for heat inactivated and opsonized (à) and heat inactivated (à) treponemes are shown.

Kupffer cells were incubated alone or with different numbers of spirochetes and the production of O_2_^- ^was determined by chemiluminescence. The treponemes, as well as phorbol myristate acetate, induced O_2_^- ^production in Kupffer cells, as evaluated by CL, while no CL signal was observed in absence of stimulating agents. The CL emission was evident immediately after the exposure to stimulating agents. As shown in Fig. 1, spirochetes induced O_2_^- ^production in KC in a dose-dependent manner. The time course study of generation of oxygen radicals showed that ROS were already detectable 20 minutes after the initiation of culture. Live as well as heat-inactivated treponemes, induced O_2_^- ^production with a peak 35 min after the addition of spirochetes. Opsonization of live treponemes did not increase O_2_^- ^production. On the contrary, opsonization of heat-inactivated spirochetes sharply increased O_2_^- ^production, with a peak observed at 35 min of incubation. The chemiluminescence signal progressively diminished during the following time of observation, being undetectable for all *T. pallidum *preparations at 180 min of incubation. The detection of ROS, performed at 4, 6 and 8 hours after the addition of the various spirochete preparations to KC, was negative (data not reported).

## Discussion

The innate immune response provides a rapid mechanism for recognizing and responding to microbial pathogens. Kupffer cells, which account for the major portion of fixed macrophages in the body and are the resident macrophages of the liver, are primarily responsible for this activity during bacteremic infections. Previous studies showed that the resistance of bacteria to the association with KC plays an important role in the occurrence of overwhelming systemic bacteremia.

In this study we have used different preparations of *T. pallidum*. Heat inactivation possibly induces the exposure of highly immunogenic internal antigens to antibodies that sharply increase the extent and rate of phagocytosis by Kupffer cells.

Our findings showed that the Kupffer cells undergo respiratory burst when they come into contact with *T. pallidum *and therefore the ROS production showed to be a primary event of KC activation occurring in the early stages of bacterial killing. All the ROS production curves of the various treponemal preparations peaked 35 minutes after the addition of bacteria to KC culture. In addition, it is important to note that the oxidative burst of *T. pallidum *phagocytosis by Kupffer cells was an early, short-lived event, which abated within 180 min of challenge. In spirochetal infections of the liver, during continual exposure of Kupffer cells to spirochetes, a continual O^-^_2 _production by Kupffer cells may sustain a deterioration of hepatic circulation and lead to the onset of liver damage.
